# Draft Genome Sequence of the Algicidal Bacterium *Mangrovimonas yunxiaonensis* Strain LY01

**DOI:** 10.1128/genomeA.01234-14

**Published:** 2014-11-26

**Authors:** Yi Li, Hong Zhu, Chongping Li, Huajun Zhang, Zhangran Chen, Wei Zheng, Hong Xu, Tianling Zheng

**Affiliations:** a State Key Laboratory for Marine Environmental Sciences and Key Laboratory of the Ministry of Education for Coastal and Wetland Ecosystem, School of Life Sciences, Xiamen University, Xiamen, China; b Key Laboratory of Marine Biogenetic Resources, Third Institute of Oceanography, State Oceanic Administration, Xiamen, China

## Abstract

*Mangrovimonas yunxiaonensis* LY01, a novel bacterium isolated from mangrove sediment, showed high algicidal effects on harmful algal blooms of *Alexandrium tamarense*. Here, we present the first draft genome sequence of this strain to further understanding of the functional genes related to algicidal activity.

## GENOME ANNOUNCEMENT

*Alexandrium tamarense* is a notorious harmful algal bloom (HAB) species, which has caused devastating economic losses and health hazards (1). Previous studies have indicated that bacteria play an important role during algal growth and influence the initiation, growth, and maintenance of HAB populations (2–4). Recently, *Mangrovimonas yunxiaonensis* LY01, a novel genus in the family *Flavobacteriaceae*, isolated from mangrove sediment of the Yunxiao mangrove National Nature Reserve, Fujian Province, China (5). The strain was deposited in the BCCM/LMG Bacteria Collection (accession number LMG 27142). Our studies have demonstrated that the strain LY01 shows high algicidal activity on *A. tamarense* (6, 7). However, until now no genome sequence related to *Mangrovimonas yunxiaonensis* has been available. To further the understanding and use of this algicidal bacterium, we determined the whole-genome sequencing of strain LY01 in this study.

The draft genome of *Mangrovimonas yunxiaonensis* LY01 was sequenced with a whole-genome shotgun strategy using an Illumina HiSeq2000 instrument. *De novo* assembly was performed using the SOAPdenovo2 alignment tool (8). Based on the assembled result of strain LY01, we found that its genome size was 2,673,510 bp, with a 39.23% G+C content and 11 contigs. The results showed that the genome of strain LY01 contained 2,468 genes. The total length of the genes was 2,435,004 bp, which makes up 91.08% of the genome. The number of tandem repeat sequences was 123. The total length of tandem repeat sequences was 28,202 bp, which make up 1.0549% of genome. The number of minisatellite DNAs was 45, the number of microsatellite DNAs was 20, the number of tRNAs was 40, and the number of rRNAs was 9. All assembled data were deposited in the NCBI nucleotide sequence database. Each gene was annotated through BlastP searches against the Swiss-Prot, COG, and KEGG databases. A total of 846 genes and 1,781 genes were assigned to COG classes and KEGG pathways, respectively.

Gene ontology (GO) searches were performed using all complete coding sequences. Results revealed that 44%, 21%, and 35% of genes were related to biological processes, cellular components, and molecular functions, respectively. From the GO category of biological processes, “cellular process” and “metabolic process” were the most dominant terms, representing 68% of genes. In the “cellular component” category, 86% of the genes were related to “cell” and “cell part.” Based on their molecular functions, 46% of the genes were identified as encoding proteins with catalytic activities and 39% of the genes were identified as encoding proteins with binding, respectively. Many of the genes (83) were identified as being associated with macromolecular complex production, and more than 20 genes for proteases or peptidases were found. Additional harmful toxins, such as insecticidal toxin complex protein (GenBank accession no. KFB00786) and hemolysin (accession no. KFB00888), were also identified. The whole-genome sequence of the novel marine strain LY01 will promote understanding of genes involved in algicidal compounds and algicidal mechanisms.

### Nucleotide sequence accession numbers.

This whole-genome shotgun project has been deposited at DDBJ/EMBL/GenBank under the accession number JPFK00000000. The first version (accession number JPFK01000000) is described in this paper. The strain *Mangrovimonas yunxiaonensis* LY01 is available from the corresponding author upon request.
